# Engaging patients, family caregivers and healthcare providers to develop metrics tailored to a palliative care population: a content validity process

**DOI:** 10.1186/s41687-025-00885-2

**Published:** 2025-05-06

**Authors:** Taylor Shorting, Madeline McCoy, Marianne Weiss, Jessica Schue, Natalie C. Ernecoff, Shirley H. Bush, Genevieve Lalumière, Jill Rice, Meaghen Hagarty, Daniel Vincent, Kirsten Wentlandt, Daniel David, Krystal Kehoe MacLeod, Vinay Kumar Mysore, Meghan Savigny, Edward Fitzgibbon, Sarina R. Isenberg

**Affiliations:** 1 Bruyère Health Research Institute, 43 Bruyère Street, Ottawa, ON K1N 5C8 Canada; 2 Marquette University, 1250 W Wisconsin Ave, Milwaukee, WI 53233 USA; 3 Johns Hopkins Bloomberg School of Public Health, 615 North Wolfe Street Baltimore, Baltimore, MD 21205 USA; 4 RAND Corporation, 4570 Fifth Avenue, Suite 600, Pittsburgh, PA 15213 USA; 5 Department of Medicine, Division of Palliative Care, University of Ottawa, 75 Laurier Ave E, Ottawa, ON K1N 6N5 Canada; 6 The Ottawa Hospital Research Institute, 1053 Carling Ave, ON Ottawa, K1Y4E9 Canada; 7 Bruyère Health, 60 Cambridge Street, Ottawa, ON K1R 7A5 Canada; 8 Regional Palliative Consultation Team (RPCT), 43 Bruyère Street, Ottawa, ON K1N 5C8 Canada; 9 The Ottawa Hospital, 501 Smyth Road, Ottawa, ON K1H 8L6 Canada; 10 University Health Network-Toronto General Hospital, 14EB-228E 200 Elizabeth Street, Toronto, ON M5G 2C4 Canada; 11 New York University College of Nursing, 433 First Avenue Room 422, NewYork, 10010 USA; 12 Department of Family Medicine, University of Ottawa, 75 Laurier Ave E, Ottawa, ON K1N 6N5 Canada; 13 Consultant, Brooklyn, New York USA; 14 Consultant, Vancouver, Canada

**Keywords:** Content validity, Palliative care, Patient-reported outcomes, Hospital discharge, discharge readiness, coping, Co-design, measurement scale

## Abstract

**Background:**

Assessment of patient readiness for hospital discharge has been advocated as an important component of discharge preparation. However, no measures focused on hospital-to-home transitions for patients receiving a palliative approach to care, or the associated difficulties in coping at home after hospital discharge, have been developed to date. Using a co-design approach, the purpose of this study was to (1) adapt two scales to a palliative care population, one of which was developed to assess readiness for the hospital-to-home transition and another developed to assess difficulty in coping post-transition and to (2) test the content validity of both scales from the perspectives of patients, family caregivers, and healthcare providers. The scales chosen for adaptation were the Readiness for Hospital Discharge Scale and Post-Discharge Coping Difficulty Scale.

**Methodology:**

The research team made initial adaptations to scale language prior to developing three parallel versions of each scale to be patient-, family caregiver-, and healthcare provider-facing. We conducted content validity testing of the items on both scales by asking each participant group to rate scale items on their usefulness, and to provide suggestions on ways items could be improved. We calculated the Item Content Validity Index and a modified Kappa statistic for each scale item, and calculated the Scale Content Validity Index for each of the three versions of the scales. Refinements were informed by qualitative feedback provided by participants during the content validity process. Final refinements were informed by members of a Patient and Family Advisory Council, and healthcare provider research team members.

**Results:**

Moderate modifications were required to the three versions of both scales. Modifications included adding items, modifying item language, and adding examples in parentheses to enhance item context. Patients, family caregivers, and healthcare providers deemed the research team’s initial modifications to the scales useful, as evidenced by each scale yielding a Scale Content Validity Index of higher than 0.5.

**Conclusion:**

The methodology provided can be used as an example of ways to engage and leverage the experiences of healthcare system users and healthcare providers throughout the outcome measures development process. The next steps will be to utilize the adapted scales as intervention outcome measures in a subsequent implementation study.

**Supplementary Information:**

The online version contains supplementary material available at 10.1186/s41687-025-00885-2.

## Introduction

People living with serious illness experience frequent hospitalizations [1]. With the goals of high-quality care and patient safety, the hospital-to-home transition serves as an opportunity to assure seamless continuity of care, and to anticipate and mitigate risks for poor experiences and outcomes [2]. For example, poorly coordinated care can lead to adverse events, avoidable rehospitalization, fragmented care, and negative implications on the physical, psychosocial, and spiritual well-being of patients and family caregivers (FCGs) (i.e., family members, friends, neighbours) [3–6]. In turn, patients often experience low satisfaction surrounding the discharge experience [3], and FCGs often feel unprepared for subsequent stages of care once the patient is discharged home [4]. 

Assessment of patient readiness for discharge has been advocated as an important component of discharge preparation [7–9]. However, no measures focused on hospital-to-home transitions, or the associated difficulties in coping at home after hospital discharge, have been developed specifically for patients who are receiving a palliative approach to care and experiencing a hospital-to-home transition [7–9]. 

Palliative populations and their FCGs encounter distinct challenges when discharged from hospital. At this stage in their care trajectory, patients are particularly vulnerable [10]. Once patients leave the hospital, many hospital-based care teams are unable to maintain active roles in patient care, thereby disrupting continuity of care. Moreover, deficiencies in discharge planning decrease quality of life for patients [11]. In many cases, patients are highly dependent on their FCGs for most, if not all, activities of daily living. FCGs are tasked with managing complex care needs [12] while facing a lack of support [11], and logistical concerns such as navigating home health systems, making legal arrangements, and coping with emotional distress [12]. Hospital-to-home discharges present unique challenges for patients receiving a palliative approach to care. At the same time, these patients often experience physical pain, depression, and a range of intense emotions, including a loss of dignity and feelings of hopelessness [10]. 

As the healthcare landscape shifts to a more person-centered approach, direct reports from patients receiving a palliative approach to care point to the imperative need to develop comprehensive assessments of treatment options and care [13]. Patient-reported outcomes (PROs) are a mechanism for capturing patients’ perceptions of their experiences of care and health outcomes. In a systematic review of existing interventions developed for patients receiving a palliative approach to care transitioning from hospital-to-home, none used a co-design approach to designing or vetting selected outcomes of importance to patients and FCGs, and only one study included in the review used PROs [14]. 

### Objectives

The objectives of this study were to adapt two scales, developed to assess readiness for the hospital-to-home transition and difficulty in coping post-transition, to a palliative care population, and to test the content validity of both scales from the perspectives of patient, FCG, and healthcare provider (HCP) participants. As a result of findings from previous co-design work [15], we determined that the two scales most suitable for adaptation were the Readiness for Hospital Discharge Scale (RHDS) and the Post Discharge Coping Difficulty Scale (PDCDS) [7, 16]. 

## Methods

### Design

Co-design is a participatory approach in which diverse stakeholders, including patients, FCGs, researchers, designers and HCPs, are engaged in intervention development while ensuring that patients and FCGs are at the forefront of the process [17]. This allows for patients and FCGs to share and integrate their lived experiences into intervention development. As noted by Munce et al., there is growing evidence that co-design can result in interventions that are more engaging, acceptable, relevant, feasible, and effective [18]. Utilizing a co-design approach ensures that outcomes effectively address the needs of patients and their FCGs. Previous studies involving patients and FCGs in the co-design process have indicated that participants experience a sense of being acknowledged [19], resulting in a positive shift towards collaboration and ownership [20]. 

In March 2022, our research team initiated a project with the goal of designing an intervention to improve the hospital-to-home transition for patients receiving palliative care and their FCGs, entitled *ACEPATH* [Advancing the Care Experience for patients receiving Palliative care as they Transition from hospital to Home]. Co-design was used to involve all stakeholders as equal collaborators in the design process to help ensure that the resulting intervention will meet their needs and is usable [21, 22]. Because the voices of patients and FCGs are essential for person-centered data collection and measurement of outcomes, our research team comprised of researchers, palliative HCPs, patients and FCGs, used a co-design process to tailor existing outcome measures to capture this population’s specific challenges and needs.

The approach to adapting the RHDS and PDCDS to be meaningful as patient and FCG-reported outcome measures was drawn from the process for contextualized measurement scale adaptation described by Ambuehl and Inauen [23]. In this study, we carried out Step 1, 2, and part of Step 3 of a 4-step process for adapting scales to a new context (i.e., the transition from hospital-to-home for patients receiving palliative care). Step 1 determined relevance and adaptations for the target population. Participants in our past-co-design work expressed that elements such as readiness for hospital discharge and coping once discharged home were important when quantifying a successful hospital to home transition [15]. We determined that the RHDS and PDCDS were most suitable for adaptation because the two scales encapsulated several elements described by participants in our previous work. The research team made several adaptations informed by co-design findings to both the RHDS and PDCDS. The purpose of Step 2 was to understand if the measurement scale and if its items could be understood by scale users. We asked participants to review the scales for relevance and to recommend modifications to scale items to ensure items were clear and relevant to the palliative care context [24]. The purpose of Step 3 was validity testing. Content validity is defined as the determination of the content representativeness or content relevance of the elements or items of an instrument to a specific population [24]. We calculated scale and item validity across patient-, FCG-, and HCP-facing versions of the RHDS and PDCDS as well as a Kappa statistic for each scale item. Following final modifications based on the content validity assessment, the research team is now ready to deploy the scales as measures in a preliminary testing of our palliative care hospital-to-home transition intervention. At that time, we will conduct reliability and additional validity testing (an additional element of Step 3) and item analysis (Step 4).

### Setting

Content adaptation and content validity testing were conducted with patients, FCGs, and palliative HCPs at the two study sites: (1) a tertiary academic hospital located in Ottawa, with a 4-bed acute palliative care unit and an interprofessional inpatient palliative care consult team, and (2) a subacute facility in Ottawa, with a 31-bed palliative care unit and physician-led inpatient palliative care consult team. Study sites were chosen to ensure our outcomes were co-designed by participants who had experienced discharges from both acute and subacute facilities. Research Ethics Board approval was obtained from Bruyère Health Research Institute as study #M16-23-033.

### Process for adapting metrics

In evaluating metrics to measure the success of our intervention, we aimed to ensure that the metrics were patient- and FCG-driven, health system relevant, and reflected insights gleaned from our previous co-design workshops. In previous co-design workshops, participants were asked to imagine their ideal transition home, and to describe the components of a successful transition [15]. In that study, the research team then synthesized the core components of signs of a successful transition; existing scales that examined the quality of hospital discharges were reviewed and evaluated as potential metrics that would later undergo adaptation.

### Step 1: Determining relevance and adaptations for the target population

In our previous co-design process, participants’ descriptions closely aligned with each of the four domains captured in the RHDS (e.g., personal status, knowledge, perceived coping ability, and expected support) [16]. The RHDS was designed to measure patient readiness for hospital discharge and has been used across a broad range of patient populations as both an outcome measure of hospitalization and as a pre-discharge assessment by the patient to facilitate evaluation and communication about discharge readiness in anticipation of discharge [7–9, 25]. Parallel forms of the RHDS for FCG self-report and for the discharging nurse assessment of a patient’s readiness for discharge are also available [25]. The PDCDS has previously been used across a broad range of adult medical-surgical patients, parents of children discharged from hospital, and postpartum women. Research has consistently demonstrated acceptable reliability and construct and predictive validity in English and translated versions of these scales [7, 16, 26, 27]. 

After determining that the RHDS and PDCDS held the most promise for adaptability to the palliative care patient population, the research team reviewed the existing items of the RHDS and PDCDS [24, 28]. We rephrased and refined the content of items of the RHDS and PDCDS to align with the experience of palliative patients transitioning from hospital to home while maintaining the key content domains. Three parallel versions of each scale were developed, one for each of the key stakeholder groups in our intervention study (patients, FCGs, HCPs).

### Steps 2 and 3: Scale understanding and validity testing

Scale understanding and content validity testing were conducted through an iterative process.

Participants included patients, FCGs, and HCPs. Patient participants consisted of individuals who would be experiencing (or had previously experienced) a hospital-to-home transition. HCPs on the research team shared a recruitment letter with their patients receiving palliative care from both study sites and requesting a response via a link to the online questionnaire on Microsoft Forms. We also invited patients who had participated in the previous co-design workshops. FCGs who had previously participated in the co-design workshops and members of our PFAC were emailed an invitation to participate by the research coordinator. The principal investigator invited project co-investigators and collaborators who identified as HCPs involved in hospital-to-home transitions at either or both sites to participate in Steps 2 and 3.

As an initial step in assessing scale understanding, we asked for input on scale content and wording from each of the three stakeholder groups for their respective version of each scale (patients, FCGs, HCPs). Patients, FCGs, and HCPs were sent their respective versions of the RHDS and PDCDS in the format of a fillable Microsoft Form via a link sent to their email. Based on their recommendations, we modified each version of the scale.

We then conducted content validity testing of the items of both scales. A minimum of four experts from each participant group were asked to rate items on the three versions of both scales on their importance in measuring discharge readiness (RHDS) and post-discharge coping difficulty (PDCDS). Participants were asked to rank each item on the 4-point Likert rating scale (1 = not useful and 4 = very useful) thereby assessing the usefulness of each item from their perspective. To clarify meaning, if participants rated items with a 1 or 2, a text box prompted participants to provide qualitative feedback on their understanding of the items and how the wording of the item could be modified to be more useful for the respective population (e.g., patients, FCGs, or HCPs). Participants were also asked to provide suggestions on additional items for the scales.

In addition to rating their respective versions of the scales, FCGs and HCPs were also asked to assess whether the items on the patient-facing scales were useful as descriptors of patient readiness for discharge and post-discharge coping using the same 4-point Likert rating scale (1 = not useful and 4 = very useful). We conducted this additional assessment because, due to their vulnerability, functional limitations, and in some cases, cognitive decline, many patients receiving a palliative approach have their HCPs or FCGs coordinate their hospital-to-home transition. As such, HCPs and FCGs may have different perspectives on what is important to consider in terms of patients’ readiness for discharge, compared to what is relevant to patients. Exploring this additional lens could provide insights for instrument modifications and for intervention design.

To quantitatively assess content validity, we generated the Item Content Validity Index [I-CVI] for each item on the scales (the proportion of respondents giving an item a relevance rating of 3-‘useful’ or 4-‘very useful’) and calculated a modified Kappa statistic for each item [24]. The modified Kappa statistic measured inter-rater reliability accounting for agreement by chance alone. We also calculated the Scale Content Validity Index [S-CVI] (the average of all I-CVIs in the scale) for the RHDS and PDCDS for each of the three versions of the scales (i.e., patient-, FCG-, and HCP-facing). We conducted our analyses using the statistical software R [29]. 

### Final refinements

The research team extracted and explored the qualitative feedback received for items with a I-CVI score of 0.5 or below, and accordingly made modifications to the identified items. After modifying the scales based on participants’ feedback from the questionnaires, we engaged our Patient and Family Advisory Council (PFAC) to review the revised patient- and FCG-facing scales and obtained their feedback through virtual discussion. PFAC members were asked whether the items included in the patient and FCG-facing scales would be useful from their respective perspectives. Our research team then met to make final refinements to the patient and FCG-facing versions of the RHDS and PDCDS to incorporate PFAC feedback. We also presented the refined HCP-facing RHDS and PDCDS to HCPs within the interdisciplinary team and asked whether the refined scales would be useful from their perspectives.

## Results

### Step 1: Determining relevance and adaptations for the target population

Across all three versions of the patient-, FCG-, and HCP-facing versions of the RHDS (PallRHDS-PT, PallRHDS-FCG, PallRHDS-HCP) and patient-, FCG-, and HCP-facing versions of the PDCDS (PallPDCDS-PT, PallPDCDS-FCG, PallPDCDS-HCP), we maintained the overarching content domains of the original scales. The initial adaptation process conducted by the research team included adding/removing items and modifying the language of existing items to be more relevant to the patient population receiving palliative care, their FCGs, and HCPs.

### Modifications made to the RHDS and PDCDS

To ensure all scale items were relevant to the hospital-to-home transition process, we made several modifications to each version of the PallRHDS and PallPDCDS. For example, we added items inquiring about how ready the patient and their FCGs are to move to their home environment with homecare and/or palliative care, and about how ready the home environment is, given the patient’s current condition. We removed items inquiring about pain, strength, energy, and physical ability. We also removed items inquiring about knowledge related to caring for oneself, personal needs, medication needs, and problems to watch for.

Table 1 shows the initial adaptations made to the PallRHDS-PT by the research team. Table 2 shows the initial adaptations made to the PallPDCDS-PT by the research team. Appendix A (Tables 1 and 2) summarizes modifications to the PallRHDS-FCG and PallPDCDS-FCG. Appendix B (Tables 1 and 2) summarizes modifications to the PallRHDS-HCP and PallPDCDS-HCP.

**Table 1:** Adaptations made to the patient-facing Readiness for Hospital Discharge Scale (PallRHDS-PT)

Original items	Initial adaptations by research team	Refinements informed by content validity	Final refinements informed by the patient and family advisory council
How physically **ready** are you to go home?	No modifications	No modifications	How **physically ready** are you to go home?
How would you describe your **pain** or **discomfort** today?	Item removed	Item removed	Item removed
How would you describe your **strength** today?	Item removed	Item removed	Item removed
How would you describe your **energy** today?	Item removed	Item removed	Item removed
How emotionally **ready** are you to go home today?	How emotionally **ready** are you to go home?	No modifications	How **emotionally ready** are you to go home?
How would you describe your **physical ability** to care for yourself today (for example, hygiene, walking, toileting)?	Item removed	Item removed	Item removed
How much do you **know about caring for yourself** after you go home?	Item removed	Item removed	Item removed
How much do you **know about** taking care of your **personal needs** (for example, hygiene, bathing, toileting, eating) after you go home?	Item removed	Item removed	Item removed
How much do you **know about** taking care of your **medical needs** (treatment, medications) after you go home)?	Item removed	Item removed	Item removed
How much do you **know about problems to watch for** after you go home?	Item removed	Item removed	Item removed
How much do you **know about who and when to call** if you have problems after you go home?	How much do you **know about who and when to call** a healthcare provider for help?	No modifications	How much do you **know about who and when to call** a healthcare provider for help once home?
How much do you **know about restrictions** (what you are allowed and not allowed to do) after you go home?	How much do you **know about the limitations or precautions** after you go home?	How much do **you know about the limitations or precautions** (for example, treatments, medications, physical limitations) after you go home?	How much do you **know about the limitations or precautions** (for example, treatments, medications, physical limitations) after you go home?
How much do you **know about what happens next** in your follow-up medical treatment plan after you go home?	No modifications	No modifications	How much do you **know about what happens next** in your follow-up medical treatment plan after you go home?
How much do you **know about services and information** available to you in your community after you go home?	How much do you **know about services and information** available in your community to you after you go home?	No modifications	How much do you **know about services** (Paid for by the government, or you/your family**) and information** available to you in your community after you go home?
How well will you be able to **handle the demands** of life at home?	How well will you and your family caregiver be able to **handle the demands** of life at home?	No modifications	How well will you be able to **handle the demands** of life at home?
How well will you be able to **perform your personal care** (for example, hygiene, bathing, toileting, eating) at home?	How well will you and your family caregiver be able to **handle your personal care** (for example, hygiene, bathing, toileting, eating) at home?	No modifications	How well will you be able to **handle your personal care** (for example, hygiene, bathing, toileting, eating) at home?
How well will you be able to **perform your medical treatments** (for example, caring for a surgical incision, respiratory treatments, exercise, rehabilitation, or taking your medications in the correct amounts and at the correct times) at home?	How well will you and your family caregiver be able to **handle your medical care** (for example, taking medication, wound dressing changes, taking care of catheter and emptying bag) at home?	No modifications	How well will you be able to **handle your medical care** (for example, taking medication, wound dressing changes, taking care of catheter and emptying bag, medical appointments, transportation needs) at home?
How much **emotional support** will you have after you go home?	No modifications	No modifications	How much **emotional support** do you expect to have after you go home?
How much **help** will you have if needed with your **personal care** after you go home?	How much **help** will you have (if needed) with your **personal care** after you go home?	No modifications	How much **help** will you have (if needed) with your **personal care** after you go home?
How much **help** will you have if needed with **household activities** (for example, cooking, cleaning, shopping, babysitting) after you go home?	How much **help** will you have (if needed) with **household activities** (for example, cooking, cleaning, shopping, babysitting) after you go home?	No modifications	How much **help** will you have (if needed) with **household activities** (for example, cooking, cleaning, shopping, babysitting) after you go home?
How much **help** will you have if needed with your **medical care** needs (treatments, medications) after you go home?	How much **help** will you have (if needed) with your **medical care** needs (treatments, medications) after you go home?	No modifications	How much **help** will you have (if needed) with your **medical care** needs (treatments, medications) after you go home
**Items Added**	How **ready** are you for moving home with **homecare and/or palliative care?**	No modifications	How **ready** are you for moving home with the support of **homecare and/or palliative care**?
	How **ready is your home environment** given your current condition (for example, is the needed equipment arranged [hospital bed in place, commode delivered, ramps installed])?	No modifications	How **ready is your home environment** given your current condition (for example, is the needed equipment arranged [hospital bed in place, commode delivered, ramps installed])?

**Table 2:** Adaptations made to the patient-facing Post-Discharge Coping Difficulty Scale (PallPDCDS-PT)

Original Items	Initial Adaptations by Research Team	Refinements Informed by Content Validity	Final Items*
How stressful has your life been? • What has been stressful?	How **stressful** has your life been since you left the hospital?	How much **stress (or anxiety)** have you felt in your life since you left the hospital?	How much **stress (or anxiety)** have you felt in your life since you left the hospital?
How much difficulty have you had with your recovery? • What has been difficult?	How much **difficulty** have you had with **handling the demands of life** since you’ve been home?	No modifications	How much **difficulty** have you had with **handling the demands of life** since you’ve been home?
How much difficulty have you had with caring for yourself? • What has been difficult?	How much **difficulty** have you had with **caring for yourself?**	How much **difficulty** have you had with **caring for yourself** (for example, eating, bathing, dressing)?	How much **difficulty** have you had with **caring for yourself** (for example, eating, bathing, dressing)?
How much difficulty have you had with managing with your medical condition? • What has been difficult?	How much **difficulty** have you encountered when **managing your medical needs?**	No modifications	How much **difficulty** have you encountered when **managing your medical needs**?
How difficult has the time been for your family members or other close persons? • What has been difficult?	How **difficult** has the time been for **your family members or other close persons?**	No modifications	Item removed
A: How much help have you needed with caring for yourself? B: How much help had you expected to need?	How much **help** have you needed with **caring for yourself?**	No modifications	How much **help** have you needed with **caring for yourself**?
How much emotional support have you needed?	No modifications	No modifications	How much **emotional support** have you needed?
How confident have you felt in your ability to care for your own needs?	How **confident** have you felt that your **care needs are being met at home?**	No modifications	How **confident** have you felt that your **care needs are being met** at home?
Have you been able to take care of your medical needs such as medications or treatments?	No modifications	No modifications	How well have you been able to **take care of your medical needs** such as medications or treatments?
How well have you adjusted to being at home since your hospitalization?	How well have you **met your expectations for your return home?**	No modifications	How well have you **met your expectations for your return home?**
**Items Added**	How **ready was your home environment** given your current condition (for example, was the needed equipment arranged [hospital bed in place, commode delivered, ramps installed])?	No modifications	How **ready was your home environment** given your current condition (for example, was the needed equipment arranged [hospital bed in place, commode delivered, ramps installed])?
	How well have you **met your expectations for your return home?**	No modifications	How well have you **met your expectations for your return home?**
	N/A	How much **support was available for emotional/physical needs** from friends, family and/or community?	How much **support has been available for emotional/physical needs** from friends, family and/or community?

*No further modifications were made to the PallPDCDS-PT after we re-engaged the patient and family advisory council to review the refined scale

### Steps 2 and 3: Scale understanding and validity testing

#### Steps 2 and 3 were conducted simultaneously

##### Participant characteristics

Four patient participants rated items on the PallRHDS-PT and PallPDCDS-PT. Five FCG participants rated items on the PallRHDS-FCG and PallPDCDS-FCG. Four HCPs participants (*n* = 3 physicians; *n* = 1 nurse) working in hospital (*n* = 3) and community (*n* = 1) settings rated items on the PallRHDS-HCP and PallPDCDS-HCP. Four FCGs and six HCPs (*n* = 4 physicians; *n* = 1 nurse, *n* = 1 ‘other’), working in hospital (*n* = 4), community (*n* = 1) or across both settings (*n* = 1) also assessed items on the PallRHDS-PT and PallPDCDS-PT.

##### Quantitative results

In content validity analysis, no items yielded an I-CVI of 0.5 or below on the PallRHDS-PT or PallRHDS-FCG; one item yielded an I-CVI of 0.5 or below on the PallRHDS-HCP. On the PallPDCDS-PT, -FCG, -HCP, an I-CVI score of 0.5 or below were obtained on no items, one item, and four items respectively. S-CVI scores were 0.97, 0.75, 0.85 for the PallRHDS-PT, -FCG, -HCP respectively, and 0.98, 0.87, and 0.73, for the PallPDCDS-PT, -FCG, -HCP respectively. Of note, S-CVI scores were lower than patients’ own ratings when FCG and HCPs rated the PallRHDS-PT (0.65 and 0.77 respectively) and the Pall-PDCDS-PT (0.64 and 0.73 respectively). Appendix C (Table 1) provides a comparison of patient, FCG, and HCP results across both scales. Appendix C (Table 2) provides a comparison of patient, FCG, and HCP participants rating items on the patient-facing RHDS and PDCDS.

##### Qualitative results

The research team extracted and reviewed qualitative feedback for items with an I-CVI score of 0.5 or below. See Appendix D (Tables 1, 2, and 3) for a summary of qualitative feedback for items with an I-CVI score of 0.5 or below. Based on the feedback from patients, FCGs and HCPs, we made additional modifications to the scales to clarify the meaning and intent of the items.

##### Changes made across all versions of the PallRHDS and PallPDCDS

Throughout all versions of the PallRHDS and PallPDCDS, we modified language to be non-dyadic (that is, not referring to the patient and FCG together in the same question) to capture the unique readiness and coping experiences of the patient and FCG. We also modified the wording to say, ‘your family member/friend’ rather than referring to ‘the patient’ in the FCG-facing scales.

##### Changes made to the PallRHDS-PT, -FCG, and -HCP

Across the various versions of the PallRHDS, we added to the parenthetical examples in several questions (e.g., limitations, precautions, self-care difficulties) to make the inquiry more specific to the palliative population. We also added additional scale items related to available supports for FCGs within the community, as well as transportation services to/from appointments and the delivery of medications.

##### FCG and HCP feedback on the PallRHDS-PT

When FCGs and HCPs were asked to rate items on the PallRHDS-PT, both groups noted that patients may not understand all facets (e.g., equipment needs, available supports) that are pertinent during the hospital-to-home transition. For example, FCGs raised concerns about items inquiring about the patient’s ability to cope at home, as well as help with personal care at home; they noted that the patient may not know answers to items of this nature prior to hospital discharge. HCPs also suggested to specify whether items inquiring about supports at home included both formal and informal supports, including support from homecare services and family members and friends. See Appendix D (Tables 4 and 5) for an overview of qualitative feedback received on the PallRHDS-PT from FCGs and HCPs.

##### Changes made to the PallPDCDS-PT, -FCG, and -HCP

Based on qualitative feedback, we made similar modifications to the PallPDCDS versions as those done for the PallRHDS; namely, changes aimed to improve understanding of the meaning and intent of the questions by adding examples for contextual clarity. Patient participants asked for scale items about the emotional and physical supports available within patients’ communities, and to modify item language to include both anxiety and stress terminology. We also removed the item inquiring about the level of difficulty experienced by other close persons to ensure item inquiry was related to the individual completing the scale. We also added an item to the PallPDCDS-PT and -FCG that inquired about supports available for emotional/physical needs from friends/family and/or community as per participants’ suggestions. Across the same two scales, we reworded an item in the PallPDCDS-PT and PallPDCDS-FCG to capture expectations met for return home. Participants highlighted the need to clarify whether items inquiring about difficulty once home should be rated within the context of extra help being received, and to specify which items are inquiring solely about the patient’s care, not the family member/friend’s own self-care.

For the PallPDCDS-HCP, HCPs emphasized the need to clarify whether item responses should consider additional supports being received at home. We found that HCP participants were concerned about the level of subjectivity HCPs would have when speaking on behalf of patient and FCGs’ experiences. Similarly, participants noted that it was likely that the HCP completing the PallPDCDS-HCP would not be the same provider who discharged the patient, thus impacting the providers’ ability to comment on expectations for returning home.

##### FCG and HCP feedback on the PallPDCDS-PT

In addition to language modifications, FCGs suggested to include items inquiring about anxiety triggers, and whether the patient feels like they could have been better prepared for their discharge home on the PallPDCDS-PT. When asked to review the PallPDCDS-PT, HCP participants highlighted the importance of listing examples on particular items (e.g., difficulty when managing medical care) to contextualize the intention of the item, and to ensure item wording is as specific as possible to avoid misinterpretation. HCPs also emphasized the need to clarify whether item responses should consider additional supports being received at home.

See Appendix D (Tables 1, 2, 3, 4, and 5) for an overview of qualitative feedback.

### Final refinements

After integrating feedback received from participants who rated the usefulness of scale items, the PFAC was asked whether the refined patient- and FCG-facing scales would be useful from their perspective. One patient and two FCGs from our PFAC provided feedback. Within the PallRHDS-PT and PallRHDS-FCG, PFAC members suggested that we modify the item inquiring about services and information to specify services paid for by the government, and services paid for by the patient/their family. The PFAC also suggested modifying the item inquiring about homecare and/or palliative care to say, ‘how ready are you for moving home with *the support of* homecare and/or palliative care?’. Within the PallRHDS-FCG, PFAC members suggested to modify the item inquiring about emotional support to say, ‘How much emotional support *do you expect to have* after your family member/friend goes home?’. The research team summarized PFAC members’ comments and suggestions to discuss them. The team reached a consensus on how to address the PFAC members’ feedback through a series of working meetings and resolved any disagreement through discussion. The researchers made no changes to the PallPDCDS-PT and PallPDCDS-FCG. HCPs on the research team reviewed the PallRHDS-HCP and PallPDCDS-HCP; we made no changes to either scale.

## Discussion

In this study, we adapted the RHDS and PDCDS to a palliative care patient population, their FCGs, and HCPs, and found that moderate modifications were required for both scales across the three different versions of each scale. Patients, FCGs, and HCPs deemed the research team’s initial modifications to the scales useful, as evidenced by S-CVI scores exceeding 0.7.

There are several notable findings worth contrasting with existing literature. We observed discrepancies between the patients’ perspectives and the perspectives of caregivers and HCPs of the item usefulness of the PallRHDS-PT and PallPDCDS-PT. This discrepancy aligns with studies that found a disconnect between patients’ perceptions of their needs versus FCGs’ and HCPs’ perceptions of the patients’ needs in their assessments of the care dimensions and how they prioritize them [30, 31]. 

A change we made to the patient- and FCG-facing scales is the removal of dyadic language suggesting that the patient and FCG would be experiencing the same levels of readiness for discharge and post-discharge coping. In the palliative care context, much of the transition coordination becomes the responsibility of the FCG. Through discussions with our various stakeholders, we determined that these different roles might yield different perceptions of readiness and coping. Other research has similarly emphasized the value of employing separate scales for patients versus FCGs, as it can help to ensure the patients’ experience is captured [32]. 

The use of PROs to evaluate complex health services interventions provides insight into the overall quality of patient and FCG experience and prioritizes outcomes that are important to them. Care setting transitions are a vulnerable time in the patient care trajectory [14], and the use of PROs to evaluate the quality of these transitions can help patients to communicate their perceptions of their health, quality of life, mental well-being and healthcare experience [33, 34]. As support from FCGs is common during hospital-to-home transitions, creating FCG-oriented versions of PROs empowers the FCG to have the opportunity to identify, measure, and address key areas that matter most to them [35]. 

Co-design is a rigorous, person-centered approach for intervention design in palliative care; however, very few studies to date have leveraged these methods to also develop and/or tailor and refine outcome metrics. Our co-design process was effective in generating three parallel versions of PROs to evaluate an intervention aimed at improving the hospital-to-home transition for people receiving palliative care.

### Strengths and limitations

Strengths of this work include the co-design approach to adapting patient-, FCG-, and HCP-reported outcome measures to the specific experience of the palliative care hospital-to-home transition. The metrics we have adapted and described in this paper will assist HCPs in anticipating discharge transition needs of patients and FCGs. The small number of respondents who participated in the content validity survey is a study limitation; however, our sample size is typical for content validity assessment [24]. Further, the generalizability of our adapted scales might be limited, as all participants engaged were from south eastern Ontario, Canada.

The implementation of a co-design approach for measure selection and refinement is indeed beneficial; however, it presents certain challenges, particularly when engaging patients, FCGs, and HCPs. From the perspective of the research team, this approach is instrumental in ensuring that our final intervention, ACEPATH, is well-aligned with the needs of its users – patients, FCGs, and HCPs. However, we encountered difficulties in engaging patients to complete the patient-facing scales. Challenges when recruiting patients during a vulnerable point in care (e.g., a hospital-to-home transition) are amplified when the patient is at end of life. We also found that FCGs experienced challenges with recall bias if they had supported a patient through a hospital-to-home transition several years prior to study engagement. Furthermore, involving a diverse sample of HCPs yielded unique perspectives that varied according to their clinical roles. It is also noteworthy that the size of our interdisciplinary research team necessitated numerous meetings to finalize the initial adaptions of the three versions of both scales.

### Next steps

The RHDS and PCDCS scales as adapted through our co-design approach to development and content validation will be used as outcome measures of the success of ACEPATH, first in a pilot implementation study, whereby we will evaluate the acceptability, appropriateness, feasibility, and fidelity of the intervention, and then in a full clinical trial. During these studies, we will continue with the process of contextualization of the RHDS and PDCDS for the palliative care population [23]; Step 3 will consist of reliability (internal consistency and test-retest) as well as construct, convergent, and predictive validity testing and Step 4 will include item response analysis.

## Conclusion

This paper provides a detailed process of leveraging co-design findings to inform the adaptation and validation of two scales to a palliative care population, and to test the content validity of both scales from the perspectives of patient, FCG, and HCP participants. The methodology provided can be used as an example of ways to engage and leverage the experiences of healthcare system users (e.g., patients and FCGs) and HCPs throughout the outcome measures development process. The next steps will be to utilize the adapted scales as outcome measures in the ACEPATH implementation study.

## Electronic supplementary material

Below is the link to the electronic supplementary material.

Supplementary Material 1

## Data Availability

N/A.

## Abbreviations

FCG: Family caregiver
HCP: Healthcare provider
I-CVI: Item-content validity index
PallPDCDS-FCG: Palliative post discharge coping difficulty scale-family caregiver
PallPDCDS-HCP: Palliative post discharge coping difficulty scale-healthcare provider
PallPDCDS-PT: Palliative post discharge coping difficulty scale-patient
PallRHDS-FCG: Palliative readiness for hospital discharge scale-family caregiver
PallRHDS-HCP: Palliative readiness for hospital discharge scale-healthcare provider
PallRHDS-PT: Palliative readiness for hospital discharge scale-patient
PDCDS: Post discharge coping difficulty scale
PFAC: Patient and family advisory council
PRO: Patient reported outcome
RHDS: Readiness for hospital discharge scale
S-CVI: Scale-item content validity index
