# Supplementary material for: Engaging patients, family caregivers and healthcare providers to develop metrics tailored to a palliative care population: a content validity process
Source: J Patient Rep Outcomes. 2025 May 6;9:47. doi: 10.1186/s41687-025-00885-2 (PMC12055745; doi:10.1186/s41687-025-00885-2)
Supplement: Supplementary file 1 — Supplementary Material 1 [file 41687_2025_885_MOESM1_ESM.docx]

**Appendix A – Adaptations Made to the PallRHDS-FCG and PallPDCDS-FCG**

Table 1: Adaptations made to the family caregiver-facing Readiness for Hospital Discharge Scale (PallRHDS-FCG).

| **Original Items** | **Initial Adaptations by Core Team** | **Refinements Informed by Content Validity** | **Final Refinements Informed by the Patient and Family Advisory Council (PFAC)** |
| --- | --- | --- | --- |
| How physically **ready** are you to go home? | How physically **ready** are you for the patient to go home? | No modifications | How **physically** **ready** are you for your family member/friend to go home? |
| How would you describe your **pain** or **discomfort** today? | Item removed | Item removed | Item removed |
| How would you describe your **strength** today? | Item removed | Item removed | Item removed |
| How would you describe your **energy** today? | Item removed | Item removed | Item removed |
| How emotionally **ready** are you to go home today? | How emotionally **ready** are you for the patient to go home? | No modifications | How **emotionally** **ready** are you for your family member/friend to go home? |
| How would you describe your **physical ability** to care for yourself today (for example, hygiene, walking, toileting)? | Item removed | Item removed | Item removed |
| How much do you **know about caring for yourself** after you go home? | Item removed | Item removed | Item removed |
| How much do you **know about** taking care of your **personal needs** (for example, hygiene, bathing, toileting, eating) after you go home? | Item removed | Item removed | Item removed |
| How much do you **know about** taking care of your **medical needs** (treatment, medications) after you go home)? | Item removed | Item removed | Item removed |
| How much do you **know about problems to watch for** after you go home? | Item removed | Item removed | Item removed |
| How much do you **know about who and when to call** if you have problems after you go home? | How much do you **know about who and when to call**a healthcare provider for help? | No modifications | How much do you **know about who and when to call**a healthcare provider for help? |
| How much do you **know about restrictions** (what you are allowed and not allowed to do) after you go home? | How much do you **know about the limitations or precautions** after the patient goes home? | How much do you **know about the limitations or precautions** (for example, treatments, medications, physical limitations) after the patient goes home? | How much do you **know about the** **limitations or precautions** (for example, treatments, medications, physical limitations) after your family member/friend goes home? |
| How much do you **know about what happens next** in your follow-up medical treatment plan after you go home? | How much do you **know about what happens next** in the patient’s follow-up medical treatment plan after you go home? | No modifications | How much do you **know about what happens next** in your family member/friend’s follow-up medical treatment plan after you go home? |
| How much do you **know about services and information** available to you in your community after you go home? | How much do you **know about services and information** available in your community to you and the patient after the patient goes home? | No modifications | How much do you **know about services** (Paid for by the government, or you/your family) **and information** available to you and your family member/friend’s in your community after your family member/friend goes home? |
| How well will you be able to **handle the demands** of life at home? | No modifications | No modifications | How well will you be able to **handle the demands** of life at home? |
| How well will you be able to **perform your personal care** (for example, hygiene, bathing, toileting, eating) at home? | How well will you be able to **handle the patient’s personal care** (for example, hygiene, bathing, toileting, eating) at home? | No modifications | How well will you be able to **handle your family member/friend’s personal care** (for example, hygiene, bathing, toileting, eating) at home? |
| How well will you be able to **perform your medical treatments** (for example, caring for a surgical incision, respiratory treatments, exercise, rehabilitation, or taking your medications in the correct amounts and at the correct times) at home? | How well will you be able to **handle the patient’s medical care** (for example, taking medication, wound dressing changes, taking care of catheter and emptying bag) at home? | How well will you be able to **handle the patient’s medical care** (for example, taking medication, wound dressing changes, taking care of catheter and emptying bag, medical appointments, transportation needs) at home? | How well will you be able to **handle your family member/friend’s** **medical care** (for example, taking medication, wound dressing changes, taking care of catheter and emptying bag, medical appointments, transportation needs) at home? |
| How much **emotional support** will you have after you go home? | How much **emotional support** will you have after the patient goes home? | No modifications | How much **emotional support** do you expect to have after your family member/friend goes home? |
| How much **help** will you have if needed with your **personal care** after you go home? | How much **help** will you have (if needed) with the patient’s **personal care** after the patient goes home? | No modifications | How much **help** will you have (as needed) with your family member/friend’s **personal care** after your family member/friend goes home? |
| How much **help** will you have if needed with **household activities** (for example, cooking, cleaning, shopping, babysitting) after you go home? | How much **help** will you have (if needed) with **household activities** (for example, cooking, cleaning, shopping, babysitting) after the patient goes home)? | No modifications | How much **help** will you have (as needed) with **household activities** related to your family member/friend (for example, cooking, cleaning, shopping, babysitting) after your family member/friend goes home? |
| How much **help** will you have if needed with your **medical care** needs (treatments, medications) after you go home? | How much **help** will you have (if needed) with the patient’s **medical care** needs (treatments, medications) after you go home? | No modifications | How much **help** will you have (as needed) with your family member/friend’s **medical care** needs (treatments, medications) after you go home? |
| **Items Added** | How **ready** are you for the patient **moving home with homecare and/or palliative care?** | No modifications | How **ready** are you for your family member/friend moving home with **homecare and/or palliative care**? |
|  | How **ready is the home environment** given their current condition (for example, is the needed equipment arranged [hospital bed in place, commode delivered, ramps installed])? | No modifications | How **ready is the home environment** given your family member/friend’s current condition (for example, is the needed equipment arranged [hospital bed in place, commode delivered, ramps installed])? |

Table 2: Adaptations made to the family caregiver-facing Post-Discharge Coping Difficulty Scale (PallPDCDS-FCG).

| **Original Items** | **Initial Adaptations by Core Team** | **Refinements Informed by Content Validity** | **Final Items*** |
| --- | --- | --- | --- |
| How stressful has your life been?   - What has been stressful? | How **stressful** has your life been since the patient left the hospital? | How much **stress (or anxiety)** have you felt in your life since the patient left the hospital? | How much **stress (or anxiety)** have you felt in your life since your family member/friend left the hospital? |
| How much difficulty have you had with your recovery?   - What has been difficult? | How much **difficulty** have you had with **handling the demands of life** since the patient has been home? | No modifications | How much **difficulty** have you had with **handling the demands of life** since your family member/friend has been home? |
| How much difficulty have you had with caring for yourself?   - What has been difficult? | How much **difficulty** have you had with **caring for yourself?** | How much **difficulty** have you had with **caring for yourself** (for example, eating, bathing, dressing)? | How much **difficulty** have you had with **caring for yourself** (for example, eating, bathing, dressing)? |
| How much difficulty have you had with managing with your medical condition?   - What has been difficult? | How much **difficulty** have you encountered when **managing the patient’s medical needs?** | No modifications | How much **difficulty** have you encountered when **managing your family member/friend’s medical needs?** |
| How difficult has the time been for your family members or other close persons?   - What has been difficult? | How **difficult** has the time been **for your family members or other close persons?** | No modifications | Item removed |
| A: How much help have you needed with caring for yourself?  B: How much help had you expected to need? | How much **help** have you needed with caring **for the patient?** | No modifications | How much **help** have you needed with caring **for your family member/friend?** |
| How much emotional support have you needed? | How much **emotional support** have you needed? | No modifications | How much **emotional support** have you needed? |
| How confident have you felt in your ability to care for your own needs? | How **confident** have you felt that the patient’s **care needs are being met** at home? | No modifications | How **confident** have you felt that your family member/friend’s **care needs are being met** at home? |
| Have you been able to take care of your medical needs such as medications or treatments? | Have you been able **to take care of the patient’s medical needs** such as medications or treatments? | No modifications | How well have you been able **to take care of your family member/friend’s medical needs** such as medications or treatments? |
| How well have you adjusted to being at home since your hospitalization? | How well have you met your **expectations for the patient’s return home?** | No modifications | How well have your **expectations for your family member/friend’s return home been met?** |
| **Items Added** | How **ready was the home environment** given the patient’s current condition (for example, was the needed equipment arranged [hospital bed in place, commode delivered, ramps installed])? | No modifications | How **ready was the home environment** given your family member/friend’s current condition (for example, was the needed equipment arranged [hospital bed in place, commode delivered, ramps installed])? |
|  | How **difficult** has the time been **for the patient?** | No modifications | Item removed |
|  | N/A | How much **support was available for emotional/physical needs from friends, family and/or community?** | How much **support has been available to you from your friends/family and/or community**? |

*No further modifications were made to the PallPDCDS-FCG after we re-engaged the PFAC to review the refined scale

**Appendix B – Adaptations Made to the PallRHDS-HCP and PallPDCDS-HCP**

Table 1: Adaptations made to the healthcare provider-facing Readiness for Hospital Discharge Scale (PallRHDS-HCP).

| **Original Items** | **Initial Adaptations by Core Team** | **Refinements Informed by Content Validity** | **Final Refinements Informed by Healthcare Providers** |
| --- | --- | --- | --- |
| How physically **ready** are you to go home? | How **physically** **ready** is the patient and/or their family/friends for the patient to go home? | No modifications | How **physically** **ready** is the patient and caregiver(s) for the patient to go home? |
| How would you describe your **pain** or **discomfort** today? | Item removed | Item removed | Item removed |
| How would you describe your **strength** today? | Item removed | Item removed | Item removed |
| How would you describe your **energy** today? | Item removed | Item removed | Item removed |
| How emotionally **ready** are you to go home today? | How **emotionally** **ready** is the patient and/or the family/friends for the patient to go home? | No modifications | How **emotionally** **ready** is the patient and caregiver(s) for the patient to go home? |
| How would you describe your **physical ability** to care for yourself today (for example, hygiene, walking, toileting)? | Item removed | Item removed | Item removed |
| How much do you **know about caring for yourself** after you go home? | Item removed | Item removed | Item removed |
| How much do you **know about** taking care of your **personal needs** (for example, hygiene, bathing, toileting, eating) after you go home? | Item removed | Item removed | Item removed |
| How much do you **know about** taking care of your **medical needs** (treatment, medications) after you go home)? | Item removed | Item removed | Item removed |
| How much do you **know about problems to watch for** after you go home? | Item removed | Item removed | Item removed |
| How much do you **know about who and when to call** if you have problems after you go home? | How much does the patient and/or their family/friends **know about who and when to call**a healthcare provider for help? | No modifications | How much does the patient and caregiver(s) **know about who and when to call** a healthcare provider for help? |
| How much do you **know about restrictions** (what you are allowed and not allowed to do) after you go home? | How much does the patient and/or their family/friends **know about the** **limitations or precautions** after the patient goes home? | How much does the patient and/or their family/friends **know about the** **limitations or precautions** (for example, Treatments, medications, physical limitations) after the patient goes home? | How much does the patient and caregiver(s) **know about the limitations or precautions** (for example, treatments, medications, physical limitations) after the patient goes home? |
| How much do you **know about what happens next** in your follow-up medical treatment plan after you go home? | How much does the patient and/or their family/friends **know about what happens next** in the patient’s follow-up medical treatment plan after you go home? | No modifications | How much does the patient and caregiver(s) **know about what happens next** in the patient’s follow-up medical treatment plan after the patient goes home? |
| How much do you **know about services and information** available to you in your community after you go home? | How much does the patient and/or their family/friends **know about services and information** available to the patient in their community after the patient goes home? | No modifications | How much does the patient and caregiver(s) **know about services** (Paid for by the government, or the patient/their family) **and information** available to the patient in their community after the patient goes home? |
| How well will you be able to **handle the demands** of life at home? | How well will the patient and/or their family/friends be able to **handle the demands**of life at home? | No modifications | How well will the patient and caregiver(s) be able to **handle the demands** of life at home? |
| How well will you be able to **perform your personal care** (for example, hygiene, bathing, toileting, eating) at home? | How well will the patient and/or their family/friends be able to **handle the patient’s personal care** (for example, hygiene, bathing, toileting, eating) at home? | No modifications | How well will the patient and caregiver(s) be able to **handle the patient’s personal care** (for example, hygiene, bathing, toileting, eating) at home? |
| How well will you be able to **perform your medical treatments** (for example, caring for a surgical incision, respiratory treatments, exercise, rehabilitation, or taking your medications in the correct amounts and at the correct times) at home? | How well will the patient and/or their family/friends be able to **handle the patient’s medical care** (for example, taking medication, wound dressing changes, taking care of catheter and emptying bag) at home? | How well will the patient and/or their family/friends be able to **handle the patient’s medical care** (for example, taking medication, wound dressing changes, taking care of catheter and emptying bag, medical appointments, transportation needs) at home? | How well will the patient and caregiver(s) be able to **handle the patient’s medical care** (for example, taking medication, wound dressing changes, taking care of catheter and emptying bag, medical appointments, transportation needs) at home? |
| How much **emotional support** will you have after you go home? | How much **emotional support** will the patient and/or their family/friends have after the patient goes home? | No modifications | How much **emotional support** will the patient and caregiver(s) have after the patient goes home? |
| How much **help** will you have if needed with your **personal care** after you go home? | How much **help** will the patient and/or their family/friends have (if needed) with the patient’s **personal care** after the patient goes home? | No modifications | How much **help** will the patient and caregiver(s) have (if needed) with the patient’s **personal care** after the patient goes home? |
| How much **help** will you have if needed with **household activities** (for example, cooking, cleaning, shopping, babysitting) after you go home? | How much **help** will the patient and/or their family/friends have (if needed) with **household activities** (for example, cooking, cleaning, shopping, babysitting) after the patient goes home? | No modifications | How much **help** will the patient and caregiver(s) have (if needed) with **household activities** (for example, cooking, cleaning, shopping, babysitting) after the patient goes home? |
| How much **help** will you have if needed with your **medical care** needs (treatments, medications) after you go home? | How much **help** will the patient and/or their family/friends have (if needed) with their **medical care** needs (treatments, medications) after the patient goes home? | No modifications | How much **help** will the patient and caregiver(s) have (if needed) with their **medical care** needs (treatments, medications) after the patient goes home? |
| **Items Added** | How **ready** is the patient and/or their family/friends for the patient to move to their home environment with **homecare and/or palliative care?** | No modifications | How **ready** is the patient and caregiver(s) for the patient to move to their home environment with **homecare and/or palliative care?** |
|  | How **ready is the home environment** given the patient’s current condition (for example, is the needed equipment arranged [hospital bed in place, commode delivered, ramps installed])? | No modifications | How **ready is the home environment** given the patient’s current condition (for example, is the needed equipment arranged [hospital bed in place, commode delivered, ramps installed])? |

Table 2: Adaptations made to the healthcare provider-facing Post-Discharge Coping Difficulty Scale (PallPDCDS-HCP).

| **Original Items** | **Initial Adaptations by Core Team** | **Refinements Informed by Content Validity** | **Final Refinements Informed by Healthcare Providers** |
| --- | --- | --- | --- |
| How stressful has your life been?   - What has been stressful? | How **stressful** has the patient and/or family/friend experience been post-discharge? | How much **stress (or anxiety)** have the patient and/or the family felt in their life since they left the hospital? | How much **stress (or anxiety)** have the patient and caregiver(s) felt in their life since they left the hospital? |
| How much difficulty have you had with your recovery?   - What has been difficult? | How much **difficulty** has the patient and/or their family/friends had with **handling the demands of life** since the patient has been home? | No modifications | How much **difficulty** has the patient and caregiver(s) had with **handling the demands of life** since the patient has been home? |
| How much difficulty have you had with caring for yourself?   - What has been difficult? | How much **difficulty** has the **patient and/or their family/friends had with caring for themself?** | How much **difficulty** has the **patient and/or their family/friends had with caring for themself** (for example, eating, bathing, dressing)? | How much **difficulty** has the patient and caregiver(s) had with **caring for the patient’s personal needs** (for example, eating, bathing, dressing)? |
| How much difficulty have you had with managing with medical condition?   - What has been difficult? | How much **difficulty** has the patient and/or their family/friends encountered when **managing the patient’s medical needs?** | No modifications | How much **difficulty** has the patient and caregiver(s) encountered when **managing the patient’s medical needs?** |
| How difficult has the time been for your family members or other close persons?   - What has been difficult? | How **difficult** has the time been for the **patient’s family members or other close persons?** | How **difficult** has the time since discharge been for the **patient’s family members or other close persons?** | How **difficult** has the time since discharge been for the **patient’s caregiver(s) or other close persons?** |
| A: How much help have you needed with caring for yourself?  B: How much help had you expected to need? | How much **help** have the patient’s family/friends needed with **caring for the patient?** | No modifications | How much **help** have the patient’s caregiver(s) needed with **caring for the patient?** |
| How much emotional support have you needed? | How much **emotional support** have the patient and/or their family/friends needed? | No modifications | How much **emotional support** have the patient and caregiver(s) needed? |
| How confident have you felt in your ability to care for your own needs? | How **confident** have you felt that the patient’s **care needs are being met** at home? | No modifications | How **confident** have the patient and caregiver(s) felt that the patient’s **care needs are being met** at home? |
| Have you been able to take care of your medical needs such as medications or treatments? | Have the patient and/or their family/friends been able to **take care of the patient’s medical needs** such as medications or treatments? | No modifications | How well have the patient and caregivers(s) been able to **take care of the patient’s medical needs** such as medications or treatments? |
| How well have you adjusted to being at home since your hospitalization? | How well have you met **your expectations for the patient’s return home?** | Item removed | Item removed |
| **Items Added** | How **ready was the home environment** given the patient’s current condition (for example, was the needed equipment arranged [hospital bed in place, commode delivered, ramps installed])? | No modifications | How **ready was the home environment** given the patient’s current condition (for example, was the needed equipment arranged [hospital bed in place, commode delivered, ramps installed])? |

**Appendix C – Content Validity Tables**

Table 1: Comparison of patient, FCG, and HCP Results

|  | Patient respondent  (PallRHDS-PT)  (n=4) | | | Family caregiver  respondent  (PallRHDS-FCG)  (n=5) | | | Healthcare provider respondent  (PallRHDS-HCP)  (n=6) | | |
| --- | --- | --- | --- | --- | --- | --- | --- | --- | --- |
| **PallRHDS^e^** | Count useful^a^ | I-CVI^b^ | Kappa*^c^ | Count useful^a^ | I-CVI^b^ | Kappa*^c^ | Count useful^a^ | I-CVI^b^ | Kappa*^c^ |
| Emotional readiness | 4 | 1.00 | 1.00 | 3 | 0.6 | 0.42 | 3 | 0.75 | 0.67 |
| Physical readiness | 4 | 1.00 | 1.00 | 4 | 0.8 | 0.76 | 3 | 0.75 | 0.67 |
| Ready for homecare/palliative care | 3 | 0.75 | 0.67 | 3 | 0.6 | 0.42 | 3 | 0.75 | 0.67 |
| Home readiness | 3 | 0.75 | 0.67 | 4 | 0.8 | 0.76 | 4 | 1.00 | 1.00 |
| Knowledge of when/who to call for help | 4 | 1.00 | 1.00 | 4 | 0.8 | 0.76 | 3 | 0.75 | 0.67 |
| Knowledge of limitations | 4 | 1.00 | 1.00 | 5 | 1 | 1 | 2 | 0.50 | 0.20 |
| Knowledge of follow up plan | 4 | 1.00 | 1.00 | 4 | 0.8 | 0.76 | 4 | 1.00 | 1.00 |
| Knowledge of services available | 4 | 1.00 | 1.00 | 4 | 0.8 | 0.76 | 3 | 0.75 | 0.67 |
| Ability to handle demands | 4 | 1.00 | 1.00 | 3 | 0.6 | 0.42 | 4 | 1.00 | 1.00 |
| Ability to perform personal care | 4 | 1.00 | 1.00 | 3 | 0.6 | 0.42 | 3 | 0.75 | 0.67 |
| Ability to handle medical care | 4 | 1.00 | 1.00 | 4 | 0.8 | 0.76 | 4 | 1.00 | 1.00 |
| Emotional support at home | 4 | 1.00 | 1.00 | 3 | 0.6 | 0.42 | 3 | 0.75 | 0.67 |
| Help with personal care at home | 4 | 1.00 | 1.00 | 4 | 0.8 | 0.76 | 4 | 1.00 | 1.00 |
| Help with household activities | 4 | 1.00 | 1.00 | 4 | 0.8 | 0.76 | 4 | 1.00 | 1.00 |
| Help with medical care at home | 4 | 1.00 | 1.00 | 4 | 0.8 | 0.76 | 4 | 1.00 | 1.00 |
| S-CVI/Ave^d^ |  | 0.97 |  |  | 0.75 |  |  | 0.85 |  |
| **PallPDCDS^f^** | Patient respondent  (PallPDCDS-PT)  (n=4) | | | Family caregiver  respondent  (PallPDCDS-FCG)  (n=5) | | | Healthcare provider respondent  (PallPDCDS-HCP)  (n=6) | | |
|  | Count useful^a^ | I-CVI^b^ | Kappa*^c^ | Count useful^a^ | I-CVI^b^ | Kappa*^c^ | Count useful^a^ | I-CVI^b^ | Kappa*^c^ |
| Stress since discharge | 4 | 1.00 | 1.00 | 5 | 1 | 1 | 2 | 0.50 | 0.20 |
| Readiness of home | 4 | 1.00 | 1.00 | 5 | 1 | 1 | 3 | 0.75 | 0.67 |
| Difficulty of demands | 4 | 1.00 | 1.00 | 5 | 1 | 1 | 3 | 0.75 | 0.67 |
| Difficulty of self-care | 4 | 1.00 | 1.00 | 5 | 1 | 1 | 2 | 0.50 | 0.20 |
| Difficulty managing medical care | 4 | 1.00 | 1.00 | 4 | 0.8 | 0.76 | 4 | 1.00 | 1.00 |
| Difficulty for patient | N/A | N/A | N/A | 4 | 0.8 | 0.76 | NA | NA | NA |
| Difficulty for family | 4 | 1.00 | 1.00 | 4 | 0.8 | 0.76 | 2 | 0.50 | 0.20 |
| Amount of help needed | 4 | 1.00 | 1.00 | 5 | 1 | 1 | 4 | 1.00 | 1.00 |
| Amount of emotional support needed | 4 | 1.00 | 1.00 | 4 | 0.8 | 0.76 | 3 | 0.75 | 0.67 |
| Confidence of care needs being met | 4 | 1.00 | 1.00 | 4 | 0.8 | 0.76 | 4 | 1.00 | 1.00 |
| Ability to care for medical needs | 4 | 1.00 | 1.00 | 5 | 1 | 1 | 4 | 1.00 | 1.00 |
| Expectations for home return | 3 | 0.75 | 0.67 | 2 | 0.4 | 0.13 | 1 | 0.25 | 0 |
| S-CVI/Ave^d^ |  | 0.98 |  |  | 0.87 |  |  | 0.73 |  |

^a^ Count of respondents responding with “Useful” or “Very Useful”.

^b^ I-CVI = The proportion of respondents giving an item a relevance rating of “Useful” or “Very Useful”.

^c^ Kappa* = Modified Kappa statistic, a measure of inter-rater reliability accounting for agreement by chance alone. The standard Kappa statistic has been modified to account for agreement on usefulness.

k* = (I-CVI – probability of chance occurrence)/(1 – probability of chance occurrence).

^d^ S-CVI/Ave = The average I-CVI.

^e^ RHDS = Readiness for Hospital Discharge Scale.

^f^ PDCDS = Post-Discharge Coping Difficulty Scale.

Table 2: Patient, FCG and HCP rating items on PT-Facing Scales

|  | Patient respondent  (PallRHDS-PT)  (n=4) | | | Family caregiver  respondent  (PallRHDS-PT)  (n=4) | | | Healthcare provider respondent  (PallRHDS-PT)  (n=6) | | |  |
| --- | --- | --- | --- | --- | --- | --- | --- | --- | --- | --- |
| **PallRHDS-PT^e^** | Count useful^a^ | I-CVI^b^ | Kappa*^c^ | Count useful^a^ | I-CVI^b^ | Kappa*^c^ | Count useful^a^ | I-CVI^b^ | Kappa*^c^ | |
| Emotional readiness | 4 | 1.00 | 1.00 | 1 | 0.25 | 0 | 3 | 0.5 | 0.27 | |
| Physical readiness | 4 | 1.00 | 1.00 | 1 | 0.25 | 0 | 4 | 0.67 | 0.56 | |
| Ready for homecare/palliative care | 3 | 0.75 | 0.67 | 1 | 0.25 | 0 | 5 | 0.83 | 0.82 | |
| Home readiness | 3 | 0.75 | 0.67 | 3 | 0.75 | 0.67 | 6 | 1.00 | 1.00 | |
| Knowledge of when/who to call for help | 4 | 1.00 | 1.00 | 4 | 1.00 | 1.00 | 6 | 1.00 | 1.00 | |
| Knowledge of limitations | 4 | 1.00 | 1.00 | 4 | 1.00 | 1.00 | 1 | 0.17 | 0.08 | |
| Knowledge of follow up plan | 4 | 1.00 | 1.00 | 4 | 1.00 | 1.00 | 5 | 0.83 | 0.82 | |
| Knowledge of services available | 4 | 1.00 | 1.00 | 3 | 0.75 | 0.67 | 5 | 0.83 | 0.82 | |
| Ability to handle demands | 4 | 1.00 | 1.00 | 2 | 0.5 | 0.2 | 6 | 1.00 | 1.00 | |
| Ability to perform personal care | 4 | 1.00 | 1.00 | 3 | 0.75 | 0.67 | 5 | 0.83 | 0.82 | |
| Ability to handle medical care | 4 | 1.00 | 1.00 | 3 | 0.75 | 0.67 | 6 | 1.00 | 1.00 | |
| Emotional support at home | 4 | 1.00 | 1.00 | 2 | 0.5 | 0.2 | 5 | 0.83 | 0.82 | |
| Help with personal care at home | 4 | 1.00 | 1.00 | 2 | 0.5 | 0.2 | 3 | 0.5 | 0.27 | |
| Help with household activities | 4 | 1.00 | 1.00 | 3 | 0.75 | 0.67 | 4 | 0.67 | 0.56 | |
| Help with medical care at home | 4 | 1.00 | 1.00 | 3 | 0.75 | 0.67 | 5 | 0.83 | 0.82 | |
| S-CVI/Ave^d^ |  | 0.97 |  |  | 0.65 |  |  | 0.77 |  | |
|  |  |  |  |  |  |  |  |  |  | |
| **PallPDCDS-PT^f^** | Patient respondent  (PallPDCDS-PT)  (n=4) | | | Family caregiver  respondent  (PallPDCDS-PT)  (n=4) | | | Healthcare provider respondent  (PallPDCDS-PT)  (n=6) | | | |
|  | Count useful^a^ | I-CVI^b^ | Kappa*^c^ | Count useful^a^ | I-CVI^b^ | Kappa*^c^ | Count useful^a^ | I-CVI^b^ | Kappa*^c^ | |
| Stress since discharge | 4 | 1.00 | 1.00 | 3 | 0.75 | 0.67 | 6 | 1.00 | 1.00 | |
| Readiness of home | 4 | 1.00 | 1.00 | 3 | 0.75 | 0.67 | 5 | 0.83 | 0.82 | |
| Difficulty of demands | 4 | 1.00 | 1.00 | 2 | 0.5 | 0.2 | 4 | 0.67 | 0.56 | |
| Difficulty of self-care | 4 | 1.00 | 1.00 | 2 | 0.5 | 0.2 | 4 | 0.67 | 0.56 | |
| Difficulty managing medical care | 4 | 1.00 | 1.00 | 3 | 0.75 | 0.67 | 3 | 0.5 | 0.27 | |
| Difficulty for family | 4 | 1.00 | 1.00 | 2 | 0.5 | 0.2 | 4 | 0.67 | 0.56 | |
| Amount of help needed | 4 | 1.00 | 1.00 | 3 | 0.75 | 0.67 | 4 | 0.67 | 0.56 | |
| Amount of emotional support needed | 4 | 1.00 | 1.00 | 3 | 0.75 | 0.67 | 6 | 1.00 | 1.00 | |
| Confidence of care needs being met | 4 | 1.00 | 1.00 | 2 | 0.5 | 0.2 | 6 | 1.00 | 1.00 | |
| Ability to care for medical needs | 4 | 1.00 | 1.00 | 2 | 0.5 | 0.2 | 4 | 0.67 | 0.56 | |
| Expectations for home return | 3 | 0.75 | 0.67 | 3 | 0.75 | 0.67 | 2 | 0.33 | 0.13 | |
| S-CVI/Ave^d^ |  | 0.98 |  |  | 0.64 |  |  | 0.73 |  | |

^a^ Count of respondents responding with “Useful” or “Very Useful”.

^b^ I-CVI = The proportion of respondents giving an item a relevance rating of “Useful” or “Very Useful”.

^c^ Kappa* = Modified Kappa statistic, a measure of inter-rater reliability accounting for agreement by chance alone. The standard Kappa statistic has been modified to account for agreement on usefulness.

k* = (I-CVI – probability of chance occurrence)/(1 – probability of chance occurrence).

^d^ S-CVI/Ave = The average I-CVI.

^e^ RHDS = Readiness for Hospital Discharge Scale.

^f^ PDCDS = Post-Discharge Coping Difficulty Scale

**Appendix D – Qualitative Data Extraction**

Table 1: Qualitative feedback received on the PallRHDS-PT and PallPDCDS-PT (Patient perspective).

*Table 1 describes patient participants’ perspectives of content that was deemed missing from the* *patient-facing readiness for hospital discharge scale and post-discharge coping difficulty scale (PallRHDS-PT and PallPDCDS-PT). Columns indicate the number of participants that rated scale item usefulness, the two scales examined, and the content deemed missing in each respective scale.*

| **Participant (n=4)** | **PallRHDS-PT** | **Missing Content** |
| --- | --- | --- |
|  |  | Is there support for the caregiver available in your community? |
|  |  | A question needs to be asked around transportation to and from appointments [and] delivery of medications. |
|  | **PallPDCDS-PT** | **Missing Content** |
|  |  | [Include an item inquiring about] emotional and physical support available in the community |
|  |  | Many caregivers and/or patients don't know who to turn to re post-discharge problems |
|  |  | It is perhaps useful to ask about anxiety, not just stress in general |

Table 2: Qualitative feedback received on the PallPDCDS-FCG.

*Table 2 provides a summary of qualitative feedback from family caregiver participants on items with a I-CVI of 0.5 or below on the family caregiver-facing PDCDS (PallPDCDS-FCG). Columns indicate the number of participants that rated scale item usefulness, the scale examined, the number of participants rating items a 3 (useful) or 4 (very useful), the I-CVI associated with the scale item, kappa statistic per item, and the qualitative feedback associated with the scale item. Content that was deemed missing from the family* *caregiver-facing PDCDS is also included. Of note, no items on the family caregiver-facing RHDS (PallRHDS-FCG) yielded an I-CVI of 0.5 or below.*

| **Participant (n=5)** | **PallPDCDS-FCG** | **Number of participants rating item 3 or 4** | **I-CVI** |  | **Kappa*** | **Qualitative Feedback** |
| --- | --- | --- | --- | --- | --- | --- |
|  | Expectations for home return | 2 | 0.4 |  | 0.13 | From personal experience, I did not know what to expect. Suggest: Did you find your expectations for the patient's return home were realistic? |
|  |  |  |  |  |  | **Missing Content** |
|  |  |  |  |  |  | What are the supports, resources, available to care for all the patient's needs when she/he returns home? |

Table 3: Qualitative feedback received on the PallRHDS-HCP and PallPDCDS-HCP.

*Table 3 provides a summary of qualitative feedback from healthcare providers on items with a I-CVI of 0.5 or below on the healthcare provider-facing RHDS and PDCDS (PallRHDS-HCP and PallPDCDS-HCP). Columns indicate the number of participants that rated scale item usefulness, the scales examined, the number of participants rating items a 3 (useful) or 4 (very useful), the I-CVI associated with the scale item, kappa statistic per item, and the qualitative feedback associated with the scale item. Overall feedback on the scales is also included.*

| **Participant (n=4)** | **PallRHDS-HCP** | **Number of participants rating item 3 or 4** | **I-CVI** | **Kappa*** | **Qualitative Feedback** |
| --- | --- | --- | --- | --- | --- |
|  | Knowledge of limitations | 2 | 0.5 | 0.2 | I think listing examples is a must here, as I would not know what this question is referring to as a healthcare provider. I am assuming medication storage, functional limitations, but not too sure |
|  | **PallPDCDS-HCP** | **Number of participants rating item 3 or 4** | **I-CVI** | **Kappa*** | **Qualitative Feedback** |
|  | Stress since discharge | 2 | 0.5 | 0.2 | Would a healthcare provider be able to accurately comment on the patient/family's subjective stress level? |
|  | Difficulty of self care | 2 | 0.5 | 0.2 | See my comments on patient questionnaire. Need to clarify whether this is in the context of the extra help being received or not. |
|  |  |  |  |  | How much difficulty has the patient him/herself and/or their family/friends had with the patient's care? (Clarify that we are asking about the patient's care, not family/friends own self-care) |
|  | Difficulty for family | 2 | 0.5 | 0.2 | Subjective, can a healthcare provider speak to this accurately? Maybe it should say "What is your perception of the level of difficulty for patient's family?” |
|  | Expectations for return home | 1 | 0.25 | 0 | How well have your expectations been met for the patient's return home? |
|  |  |  |  |  | It is unlikely that the person responding to this questionnaire is the same person that discharged the patient. Not sure if this will be a relevant question in our [community] environment. |
|  |  |  |  |  | **Overall Feedback** |
|  |  |  |  |  | From the healthcare provider perspective, I thought that a lot of the questions were asking healthcare providers to comment on the subjective experience of the patient/caregiver...e.g. stress level, difficulty level, level of support. I personally do not think I could comment on these things accurately as a hospital. However, I recognize that community MDs who see the home situation more intimately might have a different opinion |

Table 4: Qualitative feedback received on the PallRHDS-PT and PallPDCDS-PT (Family caregiver perspective).

*Table 4 provides a summary of qualitative feedback from family caregivers on items with a I-CVI of 0.5 or below on the PallRHDS-PT and PallPDCDS-PT. Columns indicate the number of participants that rated scale item usefulness, the scale examined, the number of participants rating items a 3 (useful) or 4 (very useful), the I-CVI associated with the scale item, kappa statistic per item, and the qualitative feedback associated with the scale item. Content that was deemed missing from the family* *caregiver-facing PDCDS is also included.*

| **Participant (n=4)** | **PallRHDS-PT** | **Number of participants rating item 3 or 4** | **I-CVI** | **Kappa*** | **Qualitative Feedback** |
| --- | --- | --- | --- | --- | --- |
|  | Emotional readiness | 1 | 0.25 | 0 | Do you believe that your emotional situation would improve if you returned home? |
|  |  |  |  |  | I don't think the patient really knows about how emotionally ready they are. They may want to go home, but not be clear about what that will be like. Maybe: “How much would you like to go home now?" |
|  | Physical readiness | 1 | 0.25 | 0 | Do you believe that your physical situation would improve if you were at home? |
|  |  |  |  |  | The patient may not understand what is involved physically in the transition. Maybe: "Do you feel physically able to go home now?" |
|  | Readiness for homecare/palliative care | 1 | 0.25 | 0 | Do you believe that the provisions for living at home will be adequate? |
|  |  |  |  |  | I don't think the patient really understood what this involves. Maybe "Do you understand what supports will be available when you go home such as homecare and/or palliative care?" [or] "How prepared are you for this?" |
|  |  |  |  |  | The question should be directed at the primary caregiver, more so than the patient. |
|  | Ability to handle the demands | 2 | 0.5 | 0.2 | Do you think that you and your caregiver will be able to cope? |
|  |  |  |  |  | I don't think the patient is able to know the answer to this question beforehand. Maybe: "Have you been given information about the new demands when you go home?" |
|  | Emotional support at home | 2 | 0.5 | 0.2 | Do you have family and/or friends to provide you with emotional support? |
|  |  |  |  |  | Will you have enough emotional support after you go home? |
|  | Help with personal care at home | 2 | 0.5 | 0.2 | If you require help with your personal care, do you know who would provide it? |
|  |  |  |  |  | Don't always know this beforehand. Suggest: "Will you have adequate help with your personal care after you go home?" |
|  |  |  |  |  | **Missing Content** |
|  |  |  |  |  | Do you, as a caregiver, feel sufficiently informed to undertake this task? |
|  |  |  |  |  | Do you need special transportation arrangements to get to medical appointments or tests? |
|  | **PallPDCDS-PT** | **Number of participants rating item 3 or 4** | **I-CVI** | **Kappa*** | **Qualitative Feedback** |
|  | Difficulty of demands | 2 | 0.5 | 2 | What areas have been particularly challenging since the homecoming? |
|  | Difficulty of self care | 2 | 0.5 | 0.2 | Do you feel you have been able to find a balance between caring for the patient and caring for yourself? |
|  | Difficulty for family | 2 | 0.5 | 0.2 | Are your family members able to adapt to the new situation? |
|  | Confidence of care needs being met | 2 | 0.5 | 0.2 | Do you feel that you are being looked after as well as expected? |
|  | Ability to care for medical needs | 2 | 0.5 | 0.2 | Have you needed help to be able to take care...? |
|  |  |  |  |  | **Missing Content** |
|  |  |  |  |  | Is there anything that gives you anxiety? |
|  |  |  |  |  | Do you feel you could have been better prepared? |

Table 5: Qualitative feedback received on the PallRHDS-PT and PallPDCDS-PT (Healthcare provider perspective).

*Table 5 provides a summary of qualitative feedback from healthcare providers on items with a I-CVI of 0.5 or below on the PallRHDS-PT and PallPDCDS-PT. Columns indicate the number of participants that rated scale item usefulness, the scale examined, the number of participants rating items a 3 (useful) or 4 (very useful), the I-CVI associated with the scale item, kappa statistic per item, and the qualitative feedback associated with the scale item. Overall feedback on the scales is also included.*

| **Participant (n=6)** | **PallRHDS-PT** | **Number of participants rating item 3 or 4** | **I-CVI** | **Kappa*** | **Qualitative Feedback** |
| --- | --- | --- | --- | --- | --- |
|  | Emotional readiness | 3 | 0.5 | 0.27 | I wonder if "psychologically" might be less threatening. From a practical perspective, I would say that this is a risky question, as I imagine a huge proportion of patients will not feel emotionally ready to go home, particularly in the palliative care setting. |
|  |  |  |  |  | Not sure the wording is the issue, just that being emotionally ready may mean very different things to different people... Might also just be related to how prepared |
|  | Knowledge of limitations | 1 | 0.17 | 0.08 | I believe that the current wording might be tricky for patients to interpret. Are we asking about physical limitations? Are we asking about specific precautions about care? I believe this question might not even be necessary, if we ask something more broad like "How much do you know about how you will go about your activities/care at home?" |
|  |  |  |  |  | I don't really think patients will know what this question means - could you say "Do you have a good understanding of precautions you need to take after you go home [example: medication storage, functional limitations, etc.]"... as a healthcare provider, I can't even completely think of what these would be so I think it will need clarification |
|  |  |  |  |  | Maybe you could give some examples here as you have for others (preventing falls, preventing infections, medication side effects) |
|  |  |  |  |  | I'm not sure that your average patient will easily understand this question. Can you be more specific in terms of what information that you are requesting? |
|  | Help with personal care at home | 3 | 0.5 | 0.27 | Again, is this asking about PSW home care supports? Or is this asking patients/caregivers to exclude homecare services, and answer just based on friends and family members providing support? |
|  | **PallPDCDS-PT** | **Number of participants rating item 3 or 4** | **I-CVI** | **Kappa*** | **Qualitative Feedback** |
|  | Difficulty managing medical care | 3 | 0.5 | 0.27 | Suggest to add examples similar to the previous group of questions (managing medications etc.) |
|  |  |  |  |  | Give examples? Taking your medications, treatments… |
|  | Expectations for home return | 2 | 0.33 | 0.13 | How well have your expectations been met for your return home? |
|  |  |  |  |  | What about "Has your return home gone as you expected it to"? |
|  |  |  |  |  | How well have your expectations in going home been met? |
|  |  |  |  |  | **Overall Feedback** |
|  |  |  |  |  | There were a few questions that I think would be challenging to interpret from a patient's perspective.... e.g. what is meant by limitations? Does "medical care" mean ongoing oncological treatments, or the palliative care they are receiving at home? Etc. |
|  |  |  |  |  | I also think some of the questions are ambiguous in terms of whether the response should take into account the supports arranged at home, or not. |
